# Analysis of the Phenotype Differences in Siblings with Alkaptonuria

**DOI:** 10.3390/metabo12100990

**Published:** 2022-10-19

**Authors:** Andrea Zatkova, Birgitta Olsson, Lakshminarayan R. Ranganath, Richard Imrich

**Affiliations:** 1 Biomedical Research Center, Slovak Academy of Sciences, 845 05 Bratislava, Slovakia; 2 Garriguella AB, 179 62 Ekerö, Sweden; 3 Department of Clinical Biochemistry and Metabolism, Royal Liverpool University Hospital, Liverpool L7 8XP, UK

**Keywords:** alkaptonuria, sib study, *HGD* gene, genotype-phenotype correlation, rare diseases

## Abstract

Alkaptonuria (AKU) is a rare autosomal recessive disorder caused by mutations within a gene coding for homogentisate 1,2-dioxygenase (HGD). To date, 251 different variants of this gene have been reported. The metabolic disorder in AKU leads to the accumulation of homogentisic acid (HGA), resulting in ochronosis (pigmentation of the connective tissues) and severe ochronotic spondylo-arthropathy, which usually manifests in the mid-thirties. An earlier genotype–phenotype correlation study showed no differences in serum HGA levels, absolute urinary excretion of HGA, or in the clinical symptoms between patients carrying *HGD* variants leading to 1% or >30% residual HGD activity. Still, as reported previously, the variance of the excretion of the HGA was smaller within affected siblings that share a common genotype. The present study is the first ever to systematically analyze the baseline clinical data of 24 AKU sibling pairs/groups collected in the SONIA 2 (Suitability Of Nitisinone In Alkaptonuria 2) study to evaluate phenotypical differences between patients carrying the same *HGD* genetic variants. We show that even between siblings there was considerable variability in the disease severity. This indicates that some other yet unidentified genetic, biomechanical, or environmental modifying factors may contribute to accelerated pigmentation and connective tissue damage observed in some patients.

## 1. Introduction

Until recently, painkillers and joint or aortic valve replacement surgery were the only palliative treatments available for patients suffering from the rare metabolic disorder alkaptonuria (AKU) [OMIM 203500] [1]. Based on the recently published results of the SONIA 2 (Suitability Of Nitisinone In Alkaptonuria 2) clinical study [2] performed within the framework of the EU-funded DevelopAKUre project, the European Medicines Agency (EMA) approved the use of Orfadin^®^ (nitisinone) for the treatment of adult patients with AKU. 

### 1.1. Genetic Background

AKU patients carry homozygous or compound heterozygous variants within a gene coding for homogentisate dioxygenase (HGD), a single-copy gene composed of 14 exons [3,4,5]. To date, DNA sequencing has been performed in approximately 730 AKU patients, leading to the identification of 251 different *HGD* variants that are summarized in the *HGD* mutation database (http://hgddatabase.cvtisr.sk/, accessed on 31 August 2022) [6]. The HGD protein is characterized by a delicate hexameric structure that can be easily disrupted. Missense variants represent the majority (about 65%) of identified AKU-causing DNA changes, which can influence the protein structure and function in three ways: disruption of the HGD active site, effect on the HGD monomer structure, or disruption of the interactions between monomers within a hexamer (see recent review [7]). It has been demonstrated that HGD proteins carrying different variants show diminished or near-absent enzyme activity [8,9].

### 1.2. Clinical Characterization, Age of Onset, and Disease Severity 

Due to the metabolic block caused by HGD deficiency, homogentisic acid (HGA) accumulates in AKU patients. The disease is present from birth and can be diagnosed easily in childhood, as large amounts of HGA are excreted in the urine that then darkens on standing [10]. Circulating HGA forms a dark brown ochronotic pigment that is deposited in connective tissue (a process called ochronosis), mainly in the skin, sclera, spine, and large-joint cartilage, as well as in heart valves. Starting in their early 30s, AKU patients usually suffer from early onset severe arthropathy. If not diagnosed in childhood AKU patients can often be misdiagnosed with the more commonly occurring osteoarthritis. Normally, lifespan is not shortened by AKU, but the quality of life is severely affected, mainly due to the painful destruction of the joints and spine as their mechanical properties are altered by ochronosis. This process mainly affects large weight-carrying joints and the spine, suggesting that besides diet, lifestyle issues such as hobbies, activities, and occupations that minimize joint overloading are likely to be important [1].

AKU shows high phenotype heterogeneity, and it is believed that the severity is highly dependent upon the accumulated burden of retained HGA throughout one’s life. Male patients are usually more severely affected than female patients [11,12].

A disease severity index for AKU (AKUSSI) has previously been developed [13,14] and this was used during the SONIA 2 study, together with other clinical and biochemical features, to measure disease progression and the effect of nitisinone [2]. AKUSSI incorporates multiple clinical AKU outcomes that can be described in a single score, i.e., eye and ear pigmentation, kidney and prostate stones, aortic stenosis, bone fractures, tendon/ligament/muscle ruptures, osteoarticular disease (OAD), kyphosis, scoliosis, and other disease manifestations [13,14]. Two types of AKUSSI score were used: the cAKUSSI and the mAKUSSI, where the latter does not include the pigmentation parameters. As AKU patients age, they score higher on AKUSSI, reflecting an increasing disease burden [15]. Importantly, nitisinone therapy in the SONIA 2 study slowed the increase in AKUSSI [2], and the National Alkaptonuria Center (NAC) [16] study demonstrated a reversal of ochronotic pigmentation. 

The SOFIA (Subclinical Ochronotic Features In Alkaptonuria) study (part of the DevelopAKUre project) aimed to evaluate at what age the first signs of ochronosis began and whether they were present (even at microscopic level) before the onset of overt clinical symptoms of AKU, such as joint pain [15]. Although externally visible ear ochronosis was only detected from age 34 years, pigmentation within ear biopsies was already detected in a 20-year-old female, providing evidence that ochronosis can start in AKU patients before the age of 20. Eye ochronosis increased with age and was first detected in a 22-year-old. It was also shown that TIM, a marker of MMP-mediated titin degradation that reflects a remodeling of the cardiac sarcomere, is a possible indirect marker of ochronosis of the aortic valve. The inflammation biomarkers, such as SAA and serum protein thiols, increased with age with no significant difference between AKU patients and controls. Gait analysis showed abnormal results early on, despite the lack of the change in the spine and joints in patients younger than 30 years (as shown by MRI analysis). In the SOFIA study, the quality of life appeared to deteriorate seriously from the third decade [15]. 

Occasionally, several young individuals have been reported with early signs of AKU, usually ocular ochronosis, but early arthritis has also been observed in 15-year-old patients [17,18]. Pigmentation in the eye and ear was reported in two children at age 12 and 13 [19]. Thus, it seems that ochronosis starts at an early age. However, a pediatric study is needed to assess the earliest age of onset. 

### 1.3. Genotype–Phenotype Correlation Studies

Different residual catalytic activities of the HGD proteins carrying different variants were previously observed in 2000 [9]. It was believed that such differences could lead to a varying accumulation of nonmetabolized HGA in the body, which in turn could result in differences in disease severity. However, this has not been confirmed by our recent genotype–phenotype correlations study [8], in which we used SONIA 2 baseline clinical data and compared different biochemical and clinical features in patients homozygous for the G161R variant, which showed only 1% residual HGD activity (Group A), to those homozygous for the M368V or A122V variants, which instead showed >30% residual HGD activity (Group B). The u-HGA/u-urea ratio was statistically significantly (*p* = 0.037) higher in Group A compared to Group B, indicating that Group B has retained some ability to metabolize HGA also in vivo. There was, however, no difference in s-HGA or u-HGA_24_ between the two groups, as well as no difference in eye pigmentation, bone density, or degree of spondylo-arthropathy. In addition, in both Group A and Group B, u-HGA_24_ was on average more than 10 000 times higher than normal (i.e., >30 mmol in the patients compared to <3 µmol in non-AKU subjects [20]), while the difference in u-HGA/u-urea between Group A and Group B was only about 15% [8]. Thus, even in Group B, HGA levels were very high and factors other than genotype might affect the clinical manifestations of AKU. One such factor might be the amount of dietary protein (i.e., tyrosine) intake, but also the patient’s renal function has a relevant effect, as documented, e.g., by the case of an AKU individual with diabetic nephropathy who showed plasma HGA levels threefold higher than his AKU siblings and accelerated pigmentation, whose plasma and urinary excretion of HGA decreased markedly after renal transplantation [21]. 

In agreement with these findings, Vilboux et al. [22] noted that the variance of the HGA excretion was smaller within affected siblings (common genotype) compared to the variance in the unrelated patients (different genotypes), but at the same time, even between siblings, there was considerable variability in the age of onset and severity of the ochronosis. 

Thus, the disease worsens with age because of ongoing HGA accumulation, but manifests variably in individual patients, even within the same family, suggesting that such variability does not depend solely on *HGD* mutations. This indicates that some other genetic, biomechanical, or environmental factors can contribute to connective tissue damage that, consequently, may accelerate pigmentation. 

The aim of the present study was to compare disease severity between siblings with AKU, i.e., between individuals with the same AKU mutation, in order to help identify such background factors that could account for the variable severity of AKU [23].

## 2. Materials and Methods

### 2.1. AKU Patients

Fifty-five individual AKU patients, belonging to 24 sibling pairs, or groups, from the SONIA 2 study [2] were included in the present study (details on siblings can be found in Table S1). Informed consent for data collection, analysis, and publication was obtained from all SONIA 2 patients, and all procedures were in accordance with the ethical standards of the responsible committee on human experimentation (institutional and national) and with the Declaration of Helsinki from 1975, as revised in 2000. AKU diagnosis was established based on documented elevated HGA in urine and/or the bluish-black pigmentation in connective tissue (ochronosis). The most common of the clinical manifestations, seen in more than 50% of the SONIA2 patients at the baseline visit, were OAD and pain in joints and spine, pigmentation of eye, ear and eardrum, osteopenia, and scoliosis. The *HGD* gene variants causing AKU were identified and reported previously [8] (listed in Table S1). 

### 2.2. AKUSSI Scores and Biomarkers

**Figure 1:**
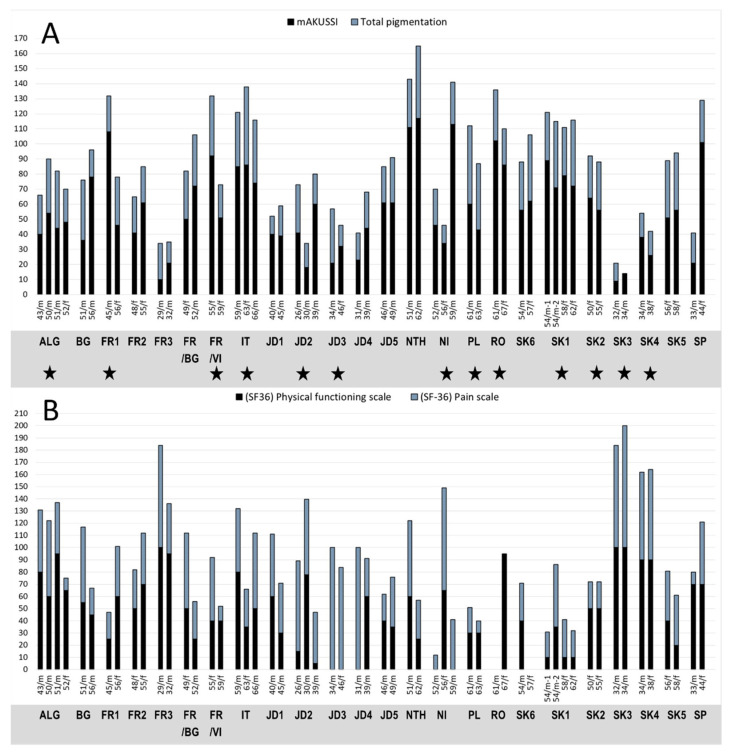
Comparison of (**A**) cAKUSSI (sum of mAKUSSI and total pigmentation), and (**B**) the physical functioning and pain scales from the self-administered SF-36 questionnaire in the siblings analyzed in our study. Age and sex are shown (m-male, f-female), the younger sibling is always to the left. Individual sibling groups are separated by a gap. Generally, an increase in cAKUSSI score indicates a worsening of the disease manifestations and this increases with age (**A**). In families marked by star the cAKUSSI score was higher in a younger sibling. In the SF-36 questionnaires (**B**), a score of 100 indicated no symptoms, whereas 0 indicated extreme symptoms; thus, the lower the score the more disability.

Baseline data from 139 patients screened in the SONIA 2 study (138 patients were randomized) were used in the analyses. Assessment of individual cAKUSSI features and measurement of biomarkers were performed as previously described [2]. cAKUSSI can be subdivided in mAKUSSI plus total pigmentation (ear pigmentation, eye pigmentation, and ear drum pigmentation scores) (as used in Figure 1A). In our present study we did not include analysis of sHGA (or urinary HGA), since it has been shown that this varies from time to time and is not (at least not on an individual level) a descriptor of disease severity. The following biomarkers, measured as exploratory endpoints during SONIA 2, were used in the present study: CTX-I (C-terminal telopeptide of type I collagen) and P1NP (procollagen type I N-terminal propeptide) are biomarkers that reflect bone resorption and bone formation, CTX-II/creatinine (C-telopeptide of type II collagen, measured in urine and normalized to excretion of creatinine), and C2M (a fragment of type II collagen released by MMP) reflect cartilage remodeling, whereas C3M (a fragment of type III collagen released by MMP) is associated with synovial inflammation and collagen degradation ((described in detail in [24] and in the submitted manuscript [25]). All markers, except CTX-II, were measured in plasma.

### 2.3. Total X ray Score

Spine radiographs, consisting of images of cervical, thoracic, and lumbosacral segments in the anteroposterior and lateral projections, were recorded using GE Optima XR646 in (General Electric Company, Boston, MA, USA) or Kodak DirectView DR7500 (Carestream Health, Rochester, NY, USA) and scored for the presence of narrowing of the intervertebral space, soft tissue calcifications in intervertebral discs and spinal ligaments, vacuum phenomena, osteophytes and/or hyperostosis, and spinal fusion as described elsewhere (manuscript submitted [26]). 

### 2.4. Total OAD Score

In order to obtain a view of the total osteoarticular disease (OAD), we calculated the score for the sum of affected joints and spinal segments (2 and 4 points, respectively, as in AKUSSI) but also added 2 points per replaced joint, assuming that they had OAD before being replaced. In AKUSSI, joint replacements are given 4 points, but were here given the same weight as a non-replaced joint with OAD. We believe that this combined score gives a good picture of the overall OAD burden.

### 2.5. Questionnaire Data

In addition to AKUSSI and some other efficacy measures, the SONIA 2 patients were also asked to fill out quality-of-life questionnaires, such as the Short Form 36 (SF-36, http://www.sf-36.org, accessed on 22 July 2016) and Knee Osteoarthritis Outcomes Score (KOOS forms, http://www.koos.nu/, accessed on 22 July 2016) [2]. We used some of the self-evaluated parameters to compare the self-reported wellbeing of studied siblings (From SF36: The Physical functioning, Physical health problems, Pain, General health perceptions, Energy fatigue, social functioning, and Emotional well-being scales. From KOOS: Pain, Symptoms, Daily living, Sport and Recreation and Quality of life). 

### 2.6. Statistical Analysis 

Statistical analyses were performed using IBM SPSS Statistics 19 (SPSS Inc., Chicago, IL, USA). All statistics carried out were post-hoc. Visit 1 (baseline) parameters including cAKUSSI, mAKUSSI, eye pigmentation, total OAD score, total X ray score, BMI, and bone and cartilage markers s-C2M, s-C3M, s-CTXI, u-CTXII/crea, and s-P1NP were correlated with age using the Pearson correlation test. SigmaPlot was used for data visualization. In all charts, the 95% confidence interval and prediction intervals are shown along with the regression lines.

## 3. Results and Discussion

Genotype–phenotype correlation studies in AKU have shown that the inter- and intra-individual variability in the HGA accumulation does not depend mainly on the specific *HGD* variants [8]. Instead, it is influenced by other factors, such as the total amount of ingested dietary proteins and renal function. Moreover, it is likely that the differences in tissue characteristics between subjects play a part in the development and progression of ochronosis. Here, we analyzed and compared baseline clinical and biochemical data from 24 AKU sibling pairs or groups from the SONIA 2 cohort in order to evaluate phenotypical differences between patients carrying the same *HGD* variants, since we believe this could be the key to understanding AKU variability.

In the first step we compared cAKUSSI scores in all sibling pairs or groups. In 13 of the 24 sibling groups, we observed that a younger sibling showed more severe AKU symptoms than an older one, as indicated mainly by the cAKUSSI score (Figure 1A), and in the majority also seen in the self-reported SF-36 physical functioning and pain scale data (Figure 1B). We thus provided the first measurable proof of clinical variability of AKU within families.

In the next step we compared the individual baseline data to look more closely at individual pairs or groups of siblings to see if there was one, or a few, symptoms that could explain the difference between them. For this purpose, we performed regression analysis for cAKUSSI and mAKUSSI scores, considering the age of individual patients and analyzed individual siblings looking for specific outliers that are more or less severely affected than their sib, or fall outside the expected values for their age group. 

By these means we confirmed the presence of real severity differences in several sibling pairs, such as FR1, FR/VI, and NI, in which the younger sibling was more severely affected than the older one (Figure 1A). We also uncovered a few other outlier sibling pairs, in which some siblings were more or sometimes less severely affected than was predicted for their age: such as the SP and NTH pairs. Generally, interesting sibling pairs fell in three categories, in which all had at least one sibling with higher scores than expected. In these sibling groups, we analyzed in more detail also the outcomes of the SF-36 and KOOS data questionnaires (shown in Figure S1).

**Figure 2:**
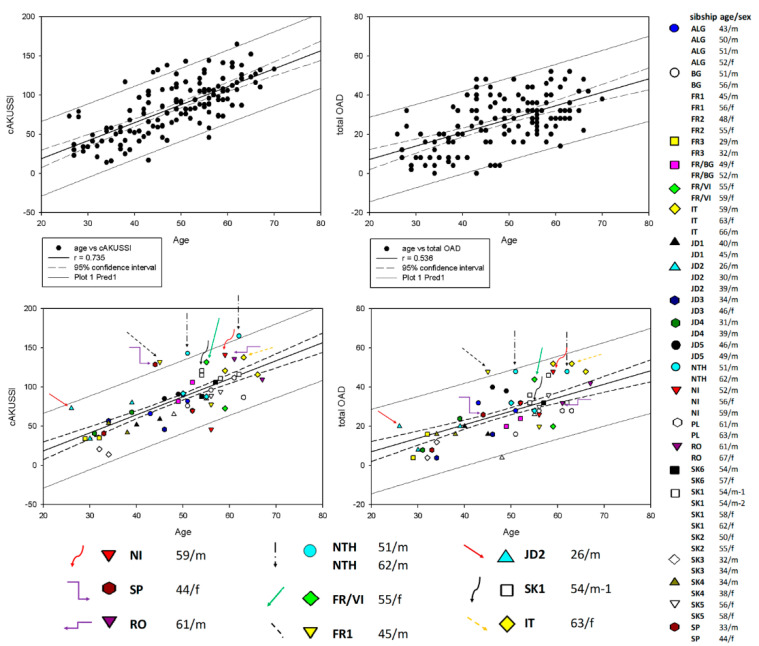
Pearson correlation of cAKUSSI (**left**) and total OAD (**right**) with age. Horizontal axis indicates the age of the patients. The upper panels show the regression analysis with all 139 SONIA 2 AKU patients. The lower panels show (on the background of the same chart) all 24 sibling pairs. The legend on the right-hand side indicates individual sibling pairs or groups, with age (in years) and sex indicated. Colored arrows indicate specific patients discussed in the text.

The first category consisted of four pairs (FR1, FR/VI, IT and RO) in which the younger sibling had higher scores than the older one (Figure 1A). For the younger sibling, cAKUSSI was above the 95% CI for their age (in case of FR1 outside of the prediction interval), and for the older it was below this interval (Figure 2).

Pair FR1 consisted of a 45-year-old male and a 56-year-old female. The brother had higher scores for cAKUSSI (Figure 1A and Figure 2), mAKUSSI, fractures, total OAD (Figure 2), and total pain score (Table S2). The two siblings did, however, have comparable scores for eye pigmentation (ochronosis), but the younger sibling had high eye pigmentation values for his age (Table S2). SF-36 and KOOS data also showed greater severity (lower score) for the brother (Figure 1B and Figure S1). As already mentioned, male patients tend to be more severely affected; thus, the differences in this sib pair may to some degree be explained by the male sex of the brother.

Pair FR/VI consisted of two females, 55 and 59 years old. The younger sister had higher scores for cAKUSSI (Figure 1A and Figure 2), mAKUSSI, eye pigmentation, ruptures, aortic valve stenosis, and total OAD (Figure 2). Interestingly, the older sister had more spinal pain and more joint replacements. She also had higher eye pigmentation. The higher cAKUSSI in the younger sister was, however, not translated into lower SF-36 and KOOS scores, as shown in Figure S1.

In the IT sibling group, consisting of three siblings, the 63-year-old sister showed a higher cAKUSSI than both her brothers, 66 and 59 years old, even though male patients are usually more severely affected than females (Figure 1A and Figure 2). The younger of the two brothers also had a slightly higher score than the older one, despite an age difference of 7 years. The female had several joint replacements, while neither brother underwent any. She also had a rupture, as did the youngest brother (Table S2). The three siblings had similar degrees of OAD, with the youngest brother showing the highest total OAD score. Low SF-36 and KOOS questionnaire scores confirmed greater severity in the female patient (Figure S1). 

In the RO sibling pair, a 61-year-old male showed cAKUSSI above the 95% CI whereas his 67-year-old sister had a score below the interval (Figure 2). The brother’s eye pigmentation, total OAD, and total X ray score were, however, within the normal ranges. He had three ruptures, while the sister had none. No other individual symptoms were remarkably different for the two siblings. No comparison of SF-36 and KOOS was possible as these data were missing for the brother.

The second category was represented by a group of three siblings (NI) in which the two youngers had cAKUSSI scores below the 95% CI while the older had cAKUSSI above the interval (Figure 1A and Figure 2). The older brother (59-years-old) had cAKUSSI and mAKUSSI that were more than twice those of his 52-year-old brother and 56-year-old sister. He had ruptures and aortic stenosis, unlike both younger siblings (Table S2). His scores were also higher for eye pigmentation, total OAD (Figure 2) and total pain (Table S2).

There was not a pronounced difference in the scores between the two younger siblings, even though the younger brother had slightly higher scores than his sister (Figure 1A and Figure 2), which may be explained by his male sex. Both had osteopenia and OAD disease (Figure 2), to about the same degree. The SF-36 data confirmed the sister’s better wellbeing (Figure 1B and Figure S1). 

The third category consisted of two pairs, in which the older sibling had the higher scores, as would be expected, but one of or both siblings also had scores that markedly exceeded the 95% CI. In the NTH pair, two brothers, 51 and 62 years old, both had high scores for cAKUSSI (Figure 1A and Figure 2), mAKUSSI, aortic stenosis, and total OAD (Figure 2, Table S2). The older brother had more eye pigmentation and a few fractures, but overall, the difference in cAKUSSI between the two corresponds was what would be expected based on their age difference. SF-36 and KOOS data indicated the same; despite the 11 years of age difference both patients showed similar profiles (Figure S1).

The second pair in this category, SP, consisted of a 33-year-old male and a 44-year-old female. Here, the older sibling had high scores for cAKUSSI (Figure 1) and mAKUSSI, while the younger had scores within or slightly below the 95% CI. These differences were mainly due to the older sister having several fractures, renal stones, and higher total OAD (Figure 2) and total pain scores (Table S2). Interestingly, the SF-36 questionnaire indicated the better wellbeing of the sister, which was, however, not confirmed by the KOOS data (Figure S1). 

The one factor in common for many cases was a high(er) score for total OAD in the sibling(s) with unexpectedly high cAKUSSI. To search for some common factors that might be responsible for, or reflect, a higher OAD in selected patients we analyzed biomarker data available from the SONIA 2 study (Table S3). 

Previous analyses showed that the levels of bone resorption (CTX-I) and cartilage degradation (C2M), as well as tissue inflammation (CRPM) markers, were elevated in AKU patients, as compared to controls [24]. In addition, this was also the case for all four exploratory biomarkers of extracellular matrix remodeling (ECMR) (i.e., C6M, VCANM, MIM, TIM). On the contrary, CTX-II, which reflects cartilage remodeling, was the only biomarker reduced in AKU patients [24]. Moreover, from recent detailed analyses of SONIA 2 data, we know that CTX-I and C2M were not significantly correlated with any of the clinical parameters (submitted manuscript [25]). 

The analyses of these biomarkers in the three sibling groups did, however, not show any association with a more severe phenotype in the cases described above (Table S3). Neither is it possible to say from these comparisons what were the reason(s) for the higher OAD scores, or for other increased symptoms in these mentioned patients.

We further looked in detail at a pair of 54-year-old monozygotic twins in the sibling group SK1. Interestingly, one of them (54/m^−1^) had higher cAKUSSI (Figure 1A and Figure 2) and mAKUSSI scores than his twin brother. However, this same patient (54/m^−1^) showed lower total OAD (Figure 2), and lower total X ray score, as well as eye pigmentation (Table S2), but, at the same time, he showed higher levels of CTX-II and P1NP, markers that reflect cartilage and bone remodeling, respectively (Table S3). The somewhat higher cAKUSSI for patient 54/m^−1^ was mainly due to the higher scores for renal and prostate stones and hearing impairment. The SF-36 questionnaire data confirmed the better wellbeing for twin 54/m^−2^ (Figure 1B and Figure S1). Their 58- and 62-years-old sisters (02-13, 02-14) had cAKUSSI within the normal range for their age (Figure 2). Since monozygotic twins share their genetic background, the phenotype differences in these brothers can be attributed only to possible epigenetic or environmental factors (including lifestyle) that have affected their development and response to the accumulated HGA burden.

Interestingly, in the three siblings JD2, a male patient of only 26 years had cAKUSSI almost as high as his 39-year-old brother (Figure 1A and Figure 2). In both siblings, cAKUSSI, mAKUSSI, eye pigmentation, and BMI were above the 95% CI. The older brother had a total OAD within the normal range, while the younger brother had a very high total OAD for his age, almost above the prediction interval (Figure 2). Their 30-year-old brother had all values except for eye pigmentation within the normal range. Eye pigmentation was above the 95% CI in all three brothers. Further analysis in the 26-year-old more severely affected patient showed values above the 95% CI for biomarkers s-CTXI, s-CTXII/crea, and s-P1NP, indicating a possible increase in bone resorption, cartilage remodeling, and inflammation processes in this patient. SF-36 confirmed the worse wellbeing of the youngest brother (in Figure S1).

## 4. Conclusions

We provided here an analysis of data which showed that there were phenotypic differences in AKU severity between siblings that share the same AKU-causing *HGD* variants, thus suggesting that there are other as yet unidentified factors that contribute to disease progression. We were not able to characterize previous loading stresses on the musculoskeletal system which could have played a part in the differences between siblings analyzed here. Differences in non-*HGD* genes and in particular those relating to connective tissue such as the collagens may be important to examine. AKU can be considered an extreme form of osteoarthrosis. As has been previously shown, studying extreme phenotypes (Extreme phenotype sampling (EPS)), can improve the understanding of more common ones. Thus, as a next step, we plan to perform whole-genome or exome analysis of selected sibling pairs as a common and effective approach for in-depth phenotyping (molecular characterization) of the disease and for evaluating the influence of non-AKU genes, including those responsible for aging and osteoarthritis susceptibility. Such data might in the future also enable the pharmacogenomic prediction of the patient’s response to planned medical therapy, such as nitisinone.

## Data Availability

Data are contained within the article or supplementary material: All clinical data were obtained during the clinical trial SONIA 2 (EU 7FP project DevelopAKUre) that has been reported extensively also in [2]. The data of selected patients presented in this study are available in Tables S1–S3. Clinical and biomarker data of the remaining sibling pairs (as listed in Table S1) are available from the corresponding author upon reasonable request.

## Supplementary Materials

The following supporting information can be downloaded at: https://www.mdpi.com/article/10.3390/metabo12100990/s1, Figure S1: Radar charts illustrating the outcomes of SF36 and KOOS questionnaires in the selected sibships described in the main text. Table S1: Complete list of individual mutations in siblings analyzed in the study. Table S2: AKUSSI scores for selected symptoms in the discussed sibships. Table S3: Biomarker concentrations in the discussed sibships.
