# Correction: Zatkova et al. Analysis of the Phenotype Differences in Siblings with Alkaptonuria. *Metabolites* 2022, *12*, 990

**DOI:** 10.3390/metabo14060339

**Published:** 2024-06-18

**Authors:** Andrea Zatkova, Birgitta Olsson, Lakshminarayan R. Ranganath, Richard Imrich

**Affiliations:** 1 Biomedical Research Center, Slovak Academy of Sciences, 845 05 Bratislava, Slovakia; 2 Garriguella AB, 179 62 Ekerö, Sweden; 3 Department of Clinical Biochemistry and Metabolism, Royal Liverpool University Hospital, Liverpool L7 8XP, UK

## Error in Figure

In the original publication [1], there was a mistake in Figure 2 as published. In the legend on the right-hand side, the signs indicating sibling pairs FR1, FR2 and SK2 were missing. The corrected Figure 2 appears below. The authors state that the scientific conclusions are unaffected. This correction was approved by the Academic Editor. The original publication has also been updated.

**Figure 2:**
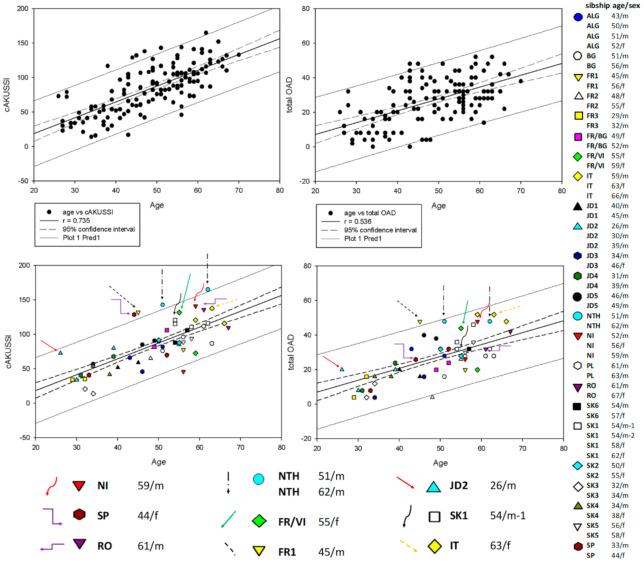
Pearson correlation of cAKUSSI (**left**) and total OAD (**right**) with age. Horizontal axis indicates the age of the patients. The upper panels show the regression analysis with all 139 SONIA 2 AKU patients. The lower panels show (on the background of the same chart) all 24 sibling pairs. The legend on the right-hand side indicates individual sibling pairs or groups, with age (in years) and sex indicated. Colored arrows indicate specific patients discussed in the text.

## Text Correction

There was an error in the original publication [1]. After the publication of the manuscript, we came to know that the twins SK1 are actually dizygotic. A correction has been made to **Results and Discussion** in ***the second to last paragraph***:

We further looked in detail at a pair of 54-year-old dizygotic twins in the sibling group SK1. Interestingly, one of them (labeled as 54/m-1) had higher cAKUSSI (Figures 1A and 2) and mAKUSSI scores than his twin brother. However, this same patient (54/m-1) showed lower total OAD (Figure 2) and a lower total X-ray score, as well as eye pigmentation (Table S2), but, at the same time, he showed higher levels of CTX-II and P1NP, markers that reflect cartilage and bone remodeling, respectively (Table S3). The somewhat higher cAKUSSI for patient 54/m-1 was mainly due to the higher scores for renal and prostate stones and hearing impairment. The SF-36 questionnaire data confirmed the better well-being of twin 54/m-2 (Figures 1B and S1). Their 58- and 62-year-old sisters had cAKUSSI within the normal range for their age (Figure 2). Like all sibs, the phenotype differences in the twin brothers can be attributed to an unknown factor from the genetic background or to possible epigenetic or environmental factors (including lifestyle) that have affected their development and response to the accumulated HGA burden.

The authors state that the scientific conclusions are unaffected. This correction was approved by the Academic Editor. The original publication has also been updated.
