# Supplementary material for: Analysis of the Phenotype Differences in Siblings with Alkaptonuria
Source: Metabolites. 2022 Oct 19;12(10):990. doi: 10.3390/metabo12100990 (PMC9611385; doi:10.3390/metabo12100990)
Supplement: Supplementary file 1 [file metabolites-12-00990-s001.zip › metabolites-1964473-supplementary.pdf]

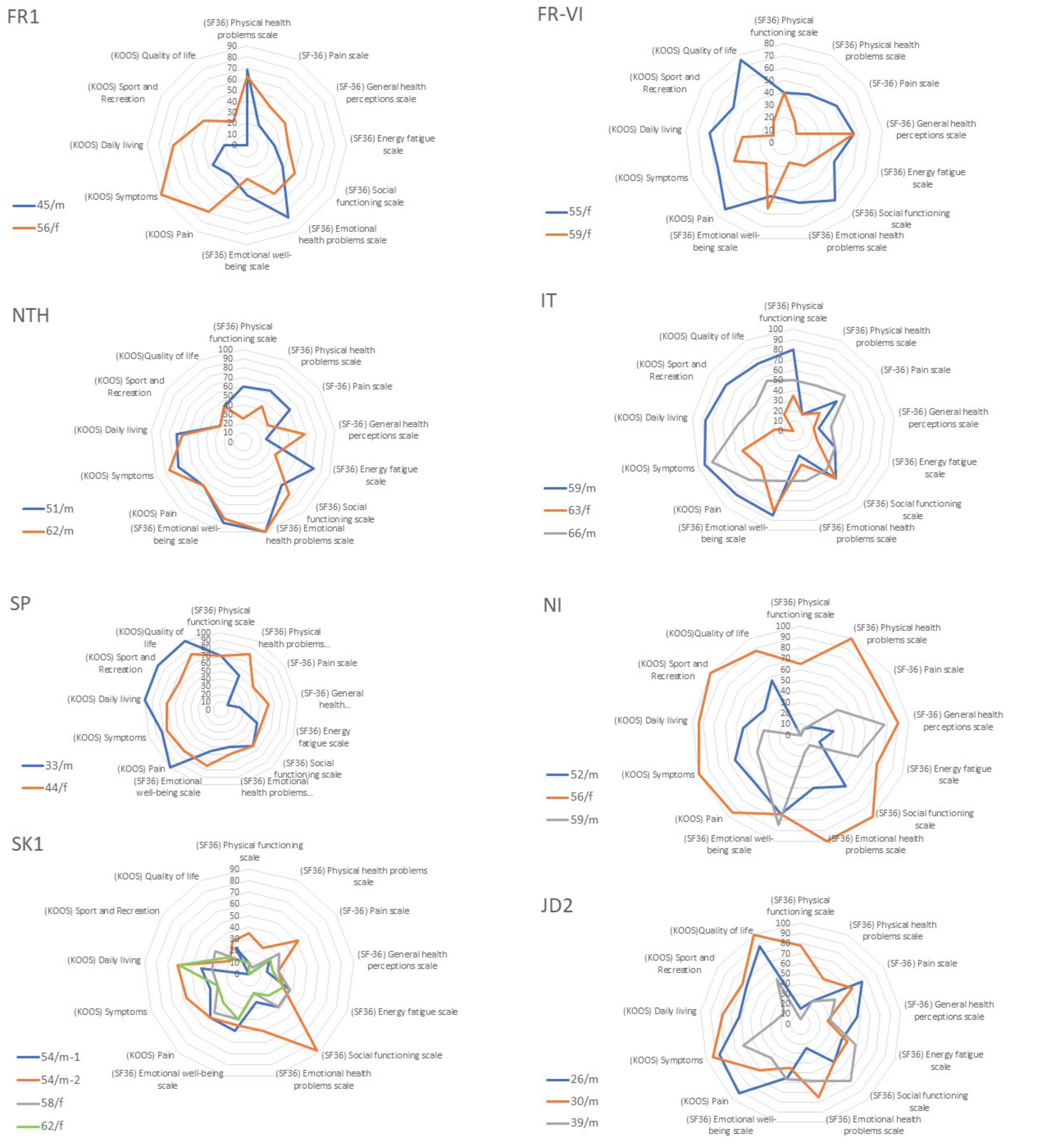

Figure S1. Radar charts illustrating the outcomes of SF36 and KOOS questionnaires in the selected sibships described in the main text. Age (years) and sex are indicated next to the colored line for each sib (m-male, f-female). In both the SF36 and KOOS questionnaires, the score of 100 indicates no symptoms, whereas 0 indicates extreme symptoms, thus, the lower the score (the smaller the circle) the more disability.

Table S1. Complete list of individual mutations in siblings analyzed in the study. Sibship code, age/sex and identified HGD variants are indicated. Variants are described at cDNA level and in one letter amino acid codes (details can be found in the HGD mutation database (hgddatabase.cvtisr.sk, accessed on 31 August 2022).

#	sibship code	age/ sex	*HGD* gene variants (NM_000187.3)	HGD protein changes (NP_000178.2)
1	ALG	43/m	c.85delC/ c.85delC	Q29fs/ Q29fs
2	ALG	50/m	c.85delC/ c.85delC	Q29fs/ Q29fs
3	ALG	51/m	c.85delC/ c.85delC	Q29fs/ Q29fs
4	ALG	52/f	c.85delC/ c.85delC	Q29fs/ Q29fs
5	BG	51/m	c.1102A>G/ c.1102A>G	M368V/ M368V
6	BG	56/m	c.1102A>G/ c.1102A>G	M368V/ M368V
7	FR1	45/m	c.1201G>C/ c.1201G>C	E401Q/ E401Q
8	FR1	56/f	c.1201G>C/ c.1201G>C	E401Q/ E401Q
9	FR2	48/f	c.647T>C/ c.481G>A	I216T/ G161R
10	FR2	55/f	c.647T>C/ c.481G>A	I216T/ G161R
11	FR3	29/m	c.647T>C/ c.1078G>C	I216T/ G360R
12	FR3	32/m	c.647T>C/ c.1078G>C	I216T/ G360R
13	FR/BG	49/f	c.688C>T/ c.1102A>G	P230S/ M368V
14	FR/BG	52/m	c.688C>T/ c.1102A>G	P230S/ M368V
15	FR/VI	55/f	c.457dupG/ c.1102A>G	D153fs/ M368V
16	FR/VI	59/f	c.457dupG/ c.1102A>G	D153fs/ M368V
17	IT	59/m	c.158G>A/ c.158G>A	R53Q/ R53Q
18	IT	63/f	c.158G>A/ c.158G>A	R53Q/ R53Q
19	IT	66/m	c.158G>A/ c.158G>A	R53Q/ R53Q
20	JD1	40/m	c.365C>T/ c.365C>T	A122V/ A122V
21	JD1	45/m	c.365C>T/ c.365C>T	A122V/ A122V
22	JD2	26/m	c.365C>T/ c.365C>T	A122V/ A122V
23	JD2	30/m	c.365C>T/ c.365C>T	A122V/ A122V
24	JD2	39/m	c.365C>T/ c.365C>T	A122V/ A122V
25	JD3	34/m	c.365C>T/ c.365C>T	A122V/ A122V
26	JD3	46/f	c.365C>T/ c.365C>T	A122V/ A122V
27	JD4	31/m	c.365C>T/ ivs1-1G>A	A122V/ ivs1-1G>A
28	JD4	39/m	c.365C>T/ ivs1-1G>A	A122V/ ivs1-1G>A
29	JD5	46/m	c.16-272_87+305del/ c.16-272_87+305del	ex2del/ ex2del
30	JD5	49/m	c.16-272_87+305del/ c.16-272_87+305del	ex2del/ ex2del
31	NTH	51/m	c.502G>A/ c.502G>A	E168K/ E168K
32	NTH	62/m	c.502G>A/ c.502G>A	E168K/ E168K
33	NI	52/m	c.1017_1019delGAGinsTA/ c.1017_1019delGAGinsTA	M339fs/ M339fs
34	NI	56/f	c.1017_1019delGAGinsTA/ c.1017_1019delGAGinsTA	M339fs/ M339fs
35	NI	59/m	c.1017_1019delGAGinsTA/ c.1017_1019delGAGinsTA	M339fs/ M339fs
36	PL	61/m	c.481G>A/ ivs1-1G>A	G161R/ ivs1-1G>A
37	PL	63/m	c.481G>A/ ivs1-1G>A	G161R/ ivs1-1G>A
38	RO	61/m	c.481G>A/ c.481G>A	G161R/ G161R
39	RO	67/f	c.481G>A/ c.481G>A	G161R/ G161R
40	SK6	54/m	c.481G>A/ c.481G>A	G161R/ G161R
41	SK6	57/f	c.481G>A/ c.481G>A	G161R/ G161R
42	SK1	54/m-1	c.481G>A/ c.481G>A	G161R/ G161R

43	SK1	54/m-2	c.481G>A/ c.481G>A	G161R/ G161R
44	SK1	58/f	c.481G>A/ c.481G>A	G161R/ G161R
45	SK1	62/f	c.481G>A/ c.481G>A	G161R/ G161R
46	SK2	50/f	c.481G>A/ c.1111dupC	G161R/ H371fs
47	SK2	55/f	c.481G>A/ c.1111dupC	G161R/ H371fs
48	SK3	32/m	c.481G>A/ c.1111dupC	G161R/ H371fs
49	SK3	34/m	c.481G>A/ c.1111dupC	G161R/ H371fs
50	SK4	34/m	c.481G>A/ c.158G>A	G161R/ R53Q
51	SK4	38/f	c.481G>A/ c.158G>A	G161R/ R53Q
52	SK5	56/f	c.808G>A/ c.1111dupC	G270R/ H371fs
53	SK5	58/f	c.808G>A/ c.1111dupC	G270R/ H371fs
54	SP	33/m	c.899T>G/ c.688C>T	V300G/ P230S
55	SP	44/f	c.899T>G/ c.688C>T	V300G/ P230S

Table S2. AKUSSI scores for selected symptoms in the discussed sibships.

Sibship	Age/sex	Category	cAKU SSI	mAKU SSI	Eye pigment	Fractures	Ruptures	Renal stones	Osteopenia	Aortic stenosis	Total OAD	Total pain	Total Xray*
FR1	45/m	1	136	112	20	24	0	4	6	4	48	14	13
	56/f		78	46	24	0	8	0	2	4	20	6	14
FR/VI	55/f	1	132	92	24	0	24	0	6	12	44	4	24
	59/f		73	51	4	0	8	0	6	4	20	9	20
IT	59/m	1	121	85	32	0	8	4	0	8	36	7	22
	63/f		138	86	32	0	8	0	-	0	28	11	21
	66/m		116	74	28	0	0	0	2	4	24	10	23
RO	61/m	1	136	102	24	0	24	4	6	4	32	18	19
	67/f		110	86	20	0	0	0	6	12	42	15	23
NI	52/m	2	70	46	8	0	0	4	2	0	32	5	18
	56/f		46	34	8	0	0	0	2	0	26	4	16
	59/m		145	117	20	0	24	8	0	8	48	12	20
NTH	51/m	3	143	111	16	0	0	4	-	10	48	11	14
	62/m		165	117	32	8	8	0	4	10	48	9	17
SP	33/m	3	41	21	8	0	0	0	0	0	8	5	1
	44/f		129	101	12	40	0	8	4	0	26	17	6
SK1	54/m-1	twins	121	89	16	0	0	16	4	4	15	15	14
	54/m-2		115	71	28	0	8	0	4	4	32	12	21
	58/f		111	79	16	0	0	0	2	8	24	15	15
	62/f		116	72	28	8	0	0	4	4	12	16	21
JD2	26/m		73	41	16	0	0	0	0	0	20	11	0
	30/m		34	18	16	0	0	0	0	0	8	6	0
	39/m		80	60	16	0	0	20	0	0	20	6	9
**Scoring**					Max. 32	8/fracture	8/rupture	4/episode	Max. 6	Max. 12	2/joint +4/sp. seg. Max. 72	1/joint +2/sp. seg. Max. 38	Max. 30

* Not included in AKUSSI; sp. seg.- spinal segment

Table S3. Biomarker concentrations in discussed sibships.

Sibship	Age/sex	Category	s-C2M (ng/ml)	u-CTX-II_crea (ng/mmol)	s-CTX-I (ng/ml)	s-P1NP (ng/ml)	s-C3M (ng/ml)	s-TIM (ng/ml)
FR1	45/m	1	0,28	945	0,325	85,8	10,7	140
	56/f		0,37	411	0,253	46,3	10,4	193
FR/VI	55/f	1	0,18	2357	0,986	141	10,9	406
	59/f		-	-	-	-	-	-
IT	59/m	1	0,36	1088	0,445	82,2	11,2	200
	63/f		0,60	1674	0,554	125	12,6	181
	66/m		0,35	808	0,293	94,9	11	130
RO	61/m	1	0,46	1376	0,616	106	9,0	330
	67/f		0,35	897	0,317	124	8,8	170
NI	52/m	2	0,62	764	0,472	68,8	8,9	372
	56/f		0,37	1154	0,469	47,1	9,3	184
	59/m		0,55	1451	0,501	111	10,3	182
NTH	51/m	3	0,55	1373	0,589	119	10,2	256
	62/m		0,22	864	0,640	99,8	13,1	394
SP	33/m	3	0,53	827	0,610	61,0	11,7	233
	44/f		0,17	388	0,270	62,5	10,2	182
SK1	54/m-1	Twins	0,30	2514	0,701	199	9,7	191
	54/m-2		0,27	1890	0,563	88,7	11,7	218
	58/f		0,23	1717	0,521	116	9,6	270
	62/f		0,37	1096	0,463	94,1	13,1	306
JD2	26/m		0,42	595	0,740	122	7,7	317
	30/m		0,30	479	0,588	104	8,6	252
	39/m		0,52	331	0,854	131	11,1	378

s- serum, u- urine
